# Remedies and Their Uses

**Published:** 1906-10-13

**Authors:** 


### Remedies and their Uses.

### Diuretics of the Purin Group.

So much has been written and said concerning the evil qualities of the purin bodies, and so many pathological conditions have been attributed to their action, that it is quite refreshing to reflect that this much abused group of substances has a real use in medicine, and that several of them have been successfully employed to relieve very serious conditions of anasarca and œdema. In order to employ these remedies scientifically and with the best results to the patient, it will be well to consider for a moment their chemical constitution and relationships, and the physiological action of the various groups of which they are composed. Such a proceeding is not only interesting in itself, but enables us to work with more accuracy and precision, as it will give an indication not only of the capabilities, but also of the limitations of these drugs, and the way in which they may be advantageously combined with other remedies. Purin itself is a somewhat complicated body containing two rings or groups, one derived from pyrimidin, and the other from a body known as imidazol.

In the following formula the larger ring belongs to pyrimidin, the smaller to imidazol, condensation having taken place with the loss of two $CH_2$ groups (indicated in square brackets):—

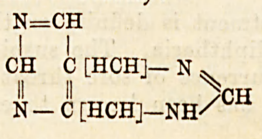

Now these two rings are composed partly of nitrogen-containing groups and partly of carbon-containing groups. Those of the latter may be oxidised, forming oxypurins, of which xanthine and uric acid are examples. The nitrogen is also capable of being altered, $CH_3$ or another radicle may be combined with any of the three atoms.

If we represent the atom groups by numbers, using Arabic numerals for the carbon and Roman for the nitrogen-containing groups, purin will be

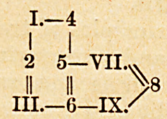

If 2 and 6 are oxidised to CO xanthine is formed, which can give rise to three dimethyl and one trimethyl compound. The dimethyl compounds are: Theobromine (III., VII. are united to $CH_3$), theophylline (I., III., $CH_3$), paraxanthine (I., VII., $CH_3$).

The trimethyl compound is caffeine (I., III., VII., $CH_3$).

Xanthine, the parent substance, is powerfully toxic, causing muscular rigors and paralysis of central origin. With the introduction of even one methyl group, some diuretic effect is produced and the toxix effect lessened. This change is not, however, absolutely quantitative. The dimethyl compounds have more diuretic action, and stimulate the muscles more markedly, while the trimethyl compound, though on the whole less potent, has more action on the central nervous system and is less diuretic. Its diuretic effect is, indeed, almost entirely due to its action on the heart, while the dimethyl compounds have a direct influence on the renal epithelium.

The two dimethyl compounds in general use are theobromine and theophylline, but these cannot be practically employed in a pure state as they are not readily absorbed. Double salts with sodium acetate and sodium salicylate have therefore been prepared, which are decomposed after absorption, allowing the purin base to exercise its characteristic function.

A double salt with Lithium salicylate, called uropherine, has also been synthesised; but it has unpleasant by-effects, and is not now employed. With theobromine the sodium salicylate compound is known as diuretin, the sodium acetate compound as agurine, the theophylline sodium compound as theocine. Recently a sodium acetate compound with theophylline has been introduced. It is called theocine-sodium-acetate.

It is remarkable that these two isomeric compounds differ somewhat in their physiological action, thus illustrating a fact frequently observed in such cases. Likewise the action of the acetic and salicylic compounds is not identical. Theocine is probably the most powerful diuretic, but it is liable to produce nausea and vomiting, and its action is not so enduring (Foà, B. M. J. Epit. 1904, 24). The sodium acetate combination is not so irritating to the stomach, and is rather more soluble. Theocine itself retains some of the excitant action on the central nervous system seen in other purin derivatives. It is therefore sometimes given in combination with hedonal. The sodium acetate combination of theobromine (agurine) is also less likely to irritate the gastric mucous membrane than the corresponding salicylate, diuretin. With all these compounds, acting as they do mainly on the renal epithelium, better effects are produced in cardiac dropsy than in dropsy dependent on the various forms of nephritis, where the kidney cells may be too damaged to respond to the drug. The diuretic effect is with all apt to be delayed, sometimes for 24 hours after the first administration. Ascites due to hepatic cirrhosis and the various inflammatory effusions into serous cavities are but little affected by drugs of this class. The four compounds may now be considered in detail, as to dosage, etc.

*Agurine* (sodium acetate and sodium theobromine).—The maximum effect as a diuretic is reached in two or three days, and is observable in healthy persons. It is a very hygroscopic powder, and when dissolved in water gradually decomposes, especially in the presence of $CO_2$. It is incompatible with acids, sugar solutions, and mucilage. It may be given in the form of a powder or in tablets. The latter are prepared by Bayer, Limited, of Elberfeld, and when dissolved yield a slightly turbid solution. This is due to an added ingredient. The powdered drug should yield a perfectly clean solution with water. The taste, in a mixture, may be covered with peppermint or cinnamon, and saccharin (but not sugar) may be added. The dose is 15 grains, three times daily.

*Diuretin* (sodium salicylate and sodium theobromine).—This substance is said to have a less unfavourable action in cases complicated by cystitis than agurine. Eight grains may be given with 8 grains of pure urea, four to eight times in the 24 hours. The solution in water does not decompose if carefully corked. It is incompatible with acids, but not with sugar solutions. It may be given in a mixture (15 grains thrice daily), the taste being covered by peppermint, or as tabloids (Burroughs and Wellcome). It may also be given in combination with digitalis or strophanthus, in cases where these drugs are indicated.

*Theocine* (sodium theophylline).—This drug, though most powerfully diuretic, has an action on the central nervous system more approaching that of caffeine. It may therefore be combined with hedonal, paraldehyde, or potassium bromide. It has not, however, any direct action on the heart. The dosage is about the same as that of caffeine (3 to 7½ grains); owing to its action on the gastric mucosa it is best to give it in small doses frequently, and always after a meal.

*Theocine sodium acetate* should be given in the same doses and with the same precautions as theocine. Its action is said to be more rapid, and it is less likely to act unfavourably on the stomach and nervous system. Both these substances have been shown to increase not only the water, but also the inorganic salts in the urine, thus aiding chloride elimination. This substance is prepared by Bayer, Limited, of Elberfeld.

